# *CLE19* expressed in the embryo regulates both cotyledon establishment and endosperm development in *Arabidopsis*

**DOI:** 10.1093/jxb/erv293

**Published:** 2015-06-12

**Authors:** Ting-Ting Xu, Shi-Chao Ren, Xiu-Fen Song, Chun-Ming Liu

**Affiliations:** ^1^Key Laboratory of Plant Molecular Physiology, Institute of Botany, Chinese Academy of Sciences, Nanxincun 20, Fragrant Hill, Beijing 100093, China; ^2^University of Chinese Academy of Sciences, Beijing 100049, China

**Keywords:** Antagonistic peptide, CLE19, cotyledon establishment, endosperm cellularization, embryo–endosperm interaction.

## Abstract

Embryo and endosperm development are two well co-ordinated developmental processes in seed formation; however, signals involved in embryo and endosperm interactions remain poorly understood. It has been shown before that CLAVATA3/ESR-RELATED 19 (CLE19) peptide is able to trigger root meristem consumption in a CLV2-dependent manner. In this study, the role of *CLE19* in *Arabidopsis* seed development was explored using antagonistic peptide technology. *CLE19* is expressed in the epidermal layers of the cotyledon primordia, hypocotyl, and root cap in the embryo. Transgenic plants carrying an antagonistic *CLE19*_*G6T*_ construct expressed under the control of *CLE19* regulatory elements exhibited a dominant seed abortion phenotype, with defective cotyledon establishment in embryos and delayed nuclear proliferation and cellularization in endosperms. Ectopic expression of *CLE19*_*G6T*_ in *Arabidopsis* under the control of an endosperm-specific *ALE1* promoter led to a similar defect in cotyledon establishment in embryos but without an evident effect on endosperm development. We therefore propose that *CLE19* may act as a mobile peptide co-ordinating embryo and endosperm development.

## Highlight

*CLE19*, as an embryo-expressed CLV3/ESR gene, regulates cotyledon establishment in embryos and nuclear proliferation and cellularization in the endosperm.

## Introduction

Double fertilization in angiosperms, where two male gametes join with the female gametophyte, initiates two parallel developmental events to produce a diploid embryo and a triploid endosperm. Embryo and endosperm development are highly co-ordinated processes (Lafon-Placette and Kohler, 2014). It has been reported previously that several endosperm-expressed genes regulate embryo development: *ABNORMAL LEAF SHAPE1* (*ALE1*) and *ZHOUPI* (*ZOU*), which are expressed in the endosperm, regulate the formation of the cuticle layer of the embryo (Tanaka *et al.*, 2001; Yang *et al.*, 2008); and *EMBRYO SURROUNDING FACTOR 1* (*ESF1*) expressed in the endosperm promotes suspensor elongation (Costa *et al.*, 2014). However, how embryo-derived factors regulate endosperm development remains elusive.

As a founding member of the *CLAVATA3 (CLV3)/EMBRYO SURROUNDING REGION (ESR)-RELATED* (*CLE*) family genes, *CLV3* acts as 12 or 13 amino acid peptide to interact with several leucine-rich repeat (LRR) receptor kinases to regulate stem cell homeostasis in shoot apical meristems (SAMs) (Clark *et al.*, 1997; Fletcher *et al.*, 1999; Kondo *et al.*, 2006; Ohyama *et al.*, 2009; Shinohara and Matsubayashi, 2015). Thirty-two *CLE* genes with diverse expression patterns have been identified in the *Arabidopsis* genome (Cock and McCormick, 2001; Oelkers *et al.*, 2008; Jun *et al.*, 2010), and among them only three have been genetically characterized. Mutation or T-DNA insertion in *CLV3* led to plants with an enlarged SAM and increased numbers of floral organs in *Arabidopsis* (Leyser and Furner, 1992). T-DNA insertion in *CLE40* led to plants with a slightly reduced root length and delayed differentiation of columella stem cells in root meristems (Hobe *et al.*, 2003; Stahl *et al.*, 2009). *CLE8* is a seed-specific *CLE* family member expressed in both embryo and endosperm in *Arabidopsis* (Sharma *et al.*, 2003), and a homozygous *cle8-1* mutant showed defective embryo and endosperm development in ~15% of the seeds produced (Fiume and Fletcher, 2012). Although *CLE41* and *CLE44* have been implicated in xylem differentiation (Ito *et al.*, 2006), *BnCLE19* in cotyledon development (Fiers *et al.*, 2004), and *ESR1* in the embryo–endosperm interaction (Opsahl-Ferstad *et al.*, 1997), since no genetic data are available for these genes, their endogenous functions remain to be elucidated. Studies performed thus far suggest a high level of redundancy among *CLE* members since mutants carrying T-DNA insertions in *CLE1*, *CLE7*, *CLE10*, *CLE16*, *CLE18*, or *CLE19* in *Arabidopsis* showed no visible phenotype (Fiers *et al.*, 2004; Jun *et al.*, 2010), and overexpression of a large group of *CLE* genes using the *cauliflower mosaic virus* (*CaMV*) *35S* promoter exhibited a similar dwarf and short-root phenotype (Fiers *et al.*, 2004; Strabala *et al.*, 2006).

*BnCLE19* is an embryo-expressed *CLE* gene that was first identified in microspore embryogenesis of *Brassica napus* (Fiers *et al.*, 2004). Transgenic *Arabidopsis* carrying the *BnCLE19* regulatory elements fused with *β-glucuronidase* (*GUS*) or *green fluorescent protein* (*GFP*) reporter genes revealed expression in cotyledon primordia in triangular-stage embryos, and the expression persisted in the epidermal layer of cotyledons during embryogenesis (Fiers *et al.*, 2004). Overexpression of *BnCLE19* or *Arabidopsis CLE19* under the control of the CaMV *35S* promoter, or treatment of *Arabidopsis* seedlings with synthetic 12–14 amino acid CLE19 peptides, led to a *CLV2*-dependent premature stem cell differentiation in root meristems (Fiers *et al.*, 2004, 2005). Suppressor screening using *RCH1-CLE19* transgenic plants led to the identification of two genetic loci, *SOL1* and *SOL2* (Casamitjana-Martínez *et al.*, 2003). *SOL1* encodes a Zn^2+^-dependent carboxypeptidase that functions to remove the C-terminal arginine residue in CLE19 processing (Casamitjana-Martínez *et al.*, 2003; Tamaki *et al.*, 2013), while *SOL2* is allelic to *CRN* that encodes an extracellular domain-free receptor-like kinase (Miwa *et al.*, 2008; Muller *et al.*, 2008). However, since the T-DNA insertional *CLE19* mutant showed no visible phenotype (Fiers *et al.*, 2004), the function of endogenous *CLE19* remains unclear.

In this study, detailed expression and functional analyses were performed in *CLE19* to elucidate its role in *Arabidopsis*. Using the recently developed antagonistic peptide technology (Song *et al.*, 2013), it was demonstrated that *CLE19*, as an embryo-specifically expressed gene, regulates both embryo and endosperm development. Transgenic plants carrying the antagonistic *CLE19*_*G6T*_ construct expressed under the control of the endogenous *CLE19* regulatory elements exhibited a dominant seed abortion phenotype, with defective cotyledon establishment and delayed endosperm development. The phenotype of altered cotyledon establishment was mimicked when *CLE19*_*G6T*_ was expressed under the control of an endosperm-specific *ALE1* promoter.

## Materials and methods

### Plant materials and growth conditions

Wild-type and transgenic *Arabidopsis thaliana* plants (Col-0) were grown either on agar plates or in soil as described previously (Song *et al.*, 2012). For transformation, 4- to 5-week-old *Arabidopsis* plants were transformed with *Agrobacterium tumefaciens* using the floral dip method (Clough and Bent, 1998).

### Molecular cloning

A *CLE19* genomic fragment containing a 1782bp 5′ upstream region (*pCLE19*), 225bp coding region, and a 1205bp 3′ downstream region (*tCLE19*) was amplified and cloned into *pDONR201* (Invitrogen) to construct the *pDONR201-pCLE19:CLE19:tCLE19* entry clone. To generate a glycine to threonine substitution (G6T) at the sixth amino acid (G6) of the CLE motif, a Fast Mutagenesis Kit (TransGen, Beijing) was used to introduce point mutations into *pDONR201-pCLE19:CLE19:tCLE19* to produce *pDONR201-pCLE19:CLE19*_*G6T*_ *:tCLE19*, which was then transferred to the *pBGWFS7* binary vector to generate *pCLE19:CLE19*_*G6T*_ *:tCLE19*. *pCLE19* and *tCLE19* were cloned into the ligation-independent cloning vectors *pPLV04* and *pPLV15* (De Rybel *et al.*, 2011) to generate *pCLE19:SV40-3XGFP:tCLE19* and *pCLE19:GUS:tCLE19*, respectively. *pWOX1:SV40-3XGFP*, *pWOX3:SV40-3XGFP*, and *pALE1:SV40-3XGFP* were made in a similar manner using *pPLV04* (De Rybel *et al.*, 2011). The *pALE1:CLE19*_*G6T*_ construct was made by replacing the *SV40-3XGFP* cassette in *pALE1:SV40-3XGFP* with the *CLE19*_*G6T*_ coding sequence.

### *In vitro* embryo culture

Siliques containing ovules with embryos at the heart-shape stage were surface-sterilized, and embryos were isolated and transferred to the top of Gamborg’s B5 basal medium (PhytoTechnology) containing 10% sucrose, 0.6% agar (type A, Sigma-Aldrich), 400 μg ml^–1^ glutamine, and 500 μg ml^–1^ inositol, and cultured as described (Liu *et al.*, 1995; Hadfi *et al.*, 1998).

### Microscopy

Siliques were fixed in pre-cooled acetone at –20°C for 1h. GUS assays were performed on fixed tissue as previously described (Fiers *et al.*, 2004). Dissected ovules were cleared in Hoyers’ solution (Berleth and Jürgens, 1993) for 2h to overnight depending on developmental stage, and were then observed under a microscope equipped with differential interference contrast (DIC). For fluorescence microscopy, ovules from transgenic plants carrying *GFP* fusion constructs were mounted with 5% glycerol and observed under a fluorescence microscope. Embryos for confocal laser scanning microscopy (CLSM) were dissected from ovules and transferred to a 9% glucose solution containing 20 μg ml^–1^ propidium iodide (PI) solution (Sigma-Aldrich) prior to imaging. ImageJ software (version 1.4.3) was used for image processing and analyses. For cytohistological analyses, periodic acid–Schiff’s reagent (PAS) staining (Baum, 2008) was performed on semi-thin sectioned ovules embedded in LR White resin (The London Resin Company).

### RT–PCR and qRT-PCR analyses

RNAs were extracted from ~300 embryos or ~100 ovules using a Plant Total RNA Isolation Kit (GeneMark, Beijing) and reverse transcribed using a First Strand cDNA Synthesis Kit (Tiangen, Beijing). Reverse transcription–PCR (RT–PCR) was performed with the resultant cDNA for analysing *EIF4A* (used as an internal control) and *CLE19* expression. Quantitative real-time PCR (qRT-PCR) was performed using a Rotor-Gene 3000 thermocycler (Corbett) with the SYBR Premix ExTaq II kit (TaKaRa, Dalian) to assess relative expression levels of *WOX1*, *WOX3*, *MEA*, *FIS2*, and *AGL62*. Expression data were normalized to *EIF4A* using the 2^–ΔΔCT^ method (Livak and Schmittgen, 2001). All primers used are listed in Supplementary Table S1 available at *JXB* online.

## Results

### Transgenic plants carrying the *pCLE19:CLE19*_*G6T*_ *:tCLE19* construct exhibited defective embryo and endosperm development

**Fig. 1.**
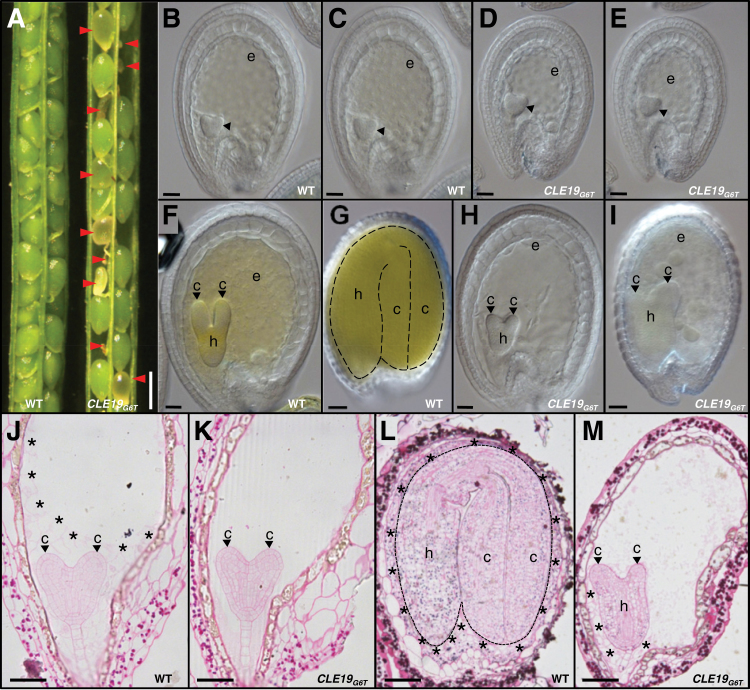
Defective embryo and endosperm development in *pCLE19:CLE19*_*G6T*_ *:tCLE19* transgenic plants. (A) Siliques from wild-type (WT) and *pCLE19:CLE19*_*G6T*_ *:tCLE19* transgenic plants (*CLE19*_*G6T*_), showing aborted ovules (indicated by arrowheads) at 12 DAP. (B–I) DIC microscopic observations of cleared ovules from wild-type (B, C, F, G) and *pCLE19:CLE19*_*G6T*_ *:tCLE19* transgenic plants (D, E, H, I) at 5 (B–E), 7 (F, H), and 12 DAP (G, I). Note the reduced sizes of embryo sacs in (D), and decreased numbers of endosperm nuclei in (E), as compared with (B) and (C), while there is no obvious defect in the embryo (indicated by arrowheads) at this stage. Delayed cotyledon formation (indicated by arrowheads) in embryos from *pCLE19:CLE19*_*G6T*_ *:tCLE19* transgenic plants at 7 (H) and 12 DAP (I), as compared with wild-type ovules at the same stages (F, G). (J–M) Cytohistological analyses of embryos and endosperms in the wild-type (J, L) and *pCLE19:CLE19*_*G6T*_ *:tCLE19* transgenic plants (K, M) at 5 (J, K) and 12 DAP (L, M), to show the delayed endosperm cellularization (indicated by asterisks) and defective cotyledon establishment (indicated by arrowheads) in ovules from *pCLE19:CLE19*_*G6T*_ *:tCLE19* transgenic plants (K, M), as compared with the wild type at the corresponding stages (J, L). c, cotyledon; h, hypocotyl; e, endosperm. Scale bars: in A=500 μm; in B–E=100 μm; in F and H=50 μm; in G and I=100 μm; and in J–M=50 μm.

It has been shown previously that *BnCLE19* is an embryo-specific gene expressed in cotyledon primordia in triangular-stage embryos, and in the epidermal layer of cotyledons in heart-shape and torpedo-stage embryos (Fiers *et al.*, 2004). In this study, the antagonistic *pCLE19:CLE19*_*G6T*_ *:tCLE19* construct (Song *et al.*, 2013) was devised to elucidate the role of *CLE19* in *Arabidopsis*. The construct consisted of a 1782bp 5′ upstream region (*pCLE19*), a 225bp coding region, and a 1205bp 3′ downstream sequence (*tCLE19*) of *CLE19*, with the conserved sixth glycine in the CLE motif substituted by threonine. The construct was transformed into *Arabidopsis* (Col-0) using the floral dip method (Clough and Bent, 1998). Among 54 independent T_1_ transgenic lines examined, 12 showed different percentages of seed abortions, and among these 12 lines, three (#1, #2, and #3) exhibited consistently high frequencies of a seed abortion phenotype (Fig. 1A). Examination of seeds at 12 days after pollination (DAP) under a dissection microscope revealed that 36.3, 33.8, and 33.4% of ovules in lines #1, #2, and #3, respectively, were aborted before maturation (Table 1). Progeny plants produced from these three lines showed a consistent seed abortion phenotype for four generations examined so far. The transgenic plants were pollinated with pollen from wild-type plants, and the resultant ovules showed similar frequencies of seed abortion, indicating that the abortion phenotype was a dominant trait. The phenotypes of these three transgenic lines were indistinguishable from one another, and transgenic line #1 was used in all subsequent studies.

**Table 1.** *Frequencies of seed abortions in* pCLE19:CLE19_G6T_:tCLE19 *and* pALE1:CLE19_G6T_ *transgenic plants*

Line	Normal	Aborted	Abortion frequency (%)
*pCLE19:CLE19*_*G6T*_ *:tCLE19*			
#1	272	155	36.3
#2	290	148	33.8
#3	289	145	33.4
*pALE1:CLE19*_*G6T*_			
#1	106	54	33.3
#2	101	40	28.4
#3	100	49	32.9

Ovules were examined under a DIC microscope after clearing using Hoyers’ solution. Embryos at the early heart-shaped stage (5 DAP) in ovules from *pCLE19:CLE19*_*G6T*_ *:tCLE19* transgenic plants were morphologically indistinguishable from those in the wild type (Fig. 1B, D). However, the size of embryo sacs in some ovules from the transgenic plants was smaller than those in the wild type at the same stage (Fig. 1B, D). In addition, reduced numbers of endosperm nuclei were often observed in these small embryo sacs (Fig. 1C, E), suggesting that endosperm development was delayed in these transgenic plants. Additionally, evident abnormal embryo development was observed in some ovules from these transgenic plants from 7 DAP onwards, with defective cotyledon formation (Fig. 1F–I, indicated by arrowheads).

Cytohistological examinations of PAS-stained semi-thin sectioned ovules confirmed that at 5 DAP embryos in *pCLE19:CLE19*_*G6T*_ *:tCLE19* transgenic plants were morphologically indistinguishable from those in the wild type (Fig. 1J, K). Strikingly, no cellularization was seen in endosperms from transgenic plants at this stage (Fig. 1K); in contrast, wild-type endosperms at the same stage showed complete cellularization in the embryo surrounding region, and partial cellularization at the periphery of the endosperm (Fig. 1J, indicated by asterisks). Even at 12 DAP, only partially cellularized endosperms were observed in aborted ovules from *pCLE19:CLE19*_*G6T*_ *:tCLE19* transgenic plants (Fig. 1L, M; indicated by asterisks), while endosperms in the wild type were cellularized completely by 8 DAP. Moreover, although cotyledon primordia were formed in aborted embryos in the transgenic plants at 12 DAP, further growth of these primordia was arrested (Fig. 1L, M; indicated by arrowheads), suggesting a defect in cotyledon establishment. In wild-type embryos, starch grains (stained dark red by PAS) were accumulated in the whole embryos by 12 DAP (Fig. 1L), while those arrested embryos from the transgenic plants exhibited a substantially lower starch accumulation in cotyledon primordia (Fig. 1M, indicated by arrowheads), suggesting that cotyledon differentiation was delayed. It should be noted that no obvious defect was observed in the hypocotyl and the root tip regions (Fig. 1I, M). Thus, expression of *CLE19*_*G6T*_ under the control of *CLE19* regulatory elements in *Arabidopsis* led to defective cotyledon establishment and delayed endosperm nuclear proliferation and cellularization.

### Expression of *CLE19* in seeds is confined to the embryo

**Fig. 2.**
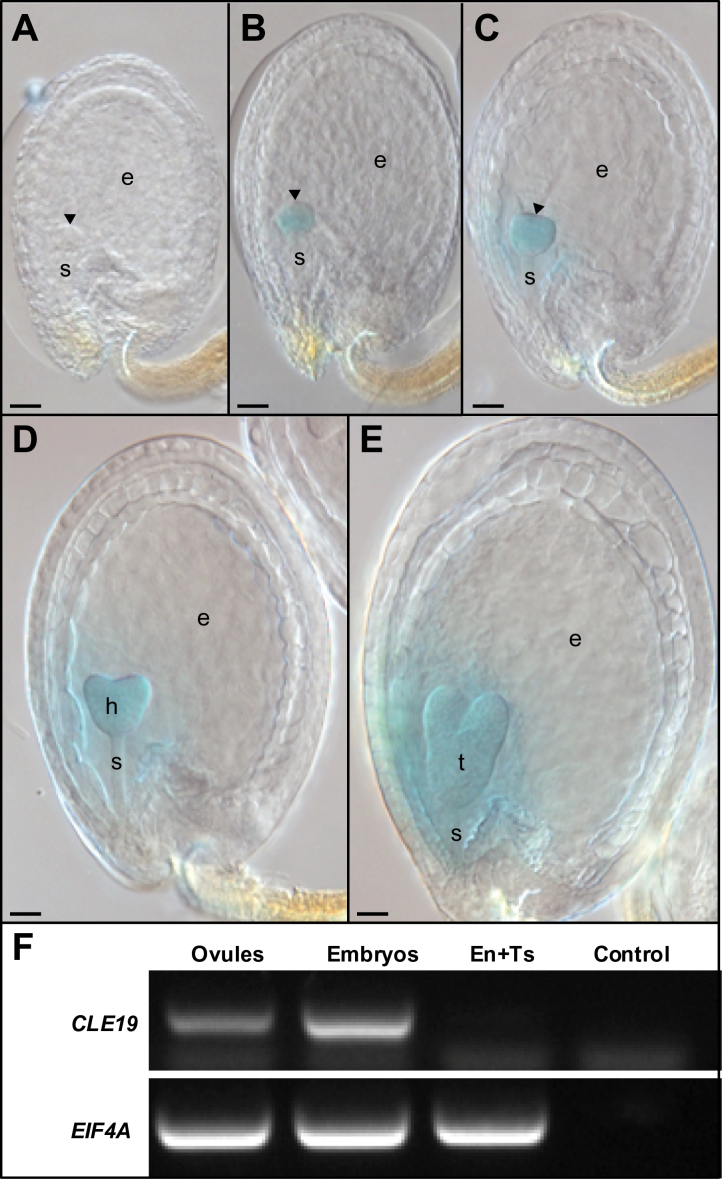
Embryo-specific expression of *CLE19* during seed development. (A–E) *GUS* expression in seeds excised from *pCLE19:GUS:tCLE19* transgenic plants. Note that GUS staining was first detected in late globular-stage embryos (B), and persisted in the triangular (C), heart-shaped (D), and torpedo-stage embryos (E), while no *GUS* expression was observed in the early globular embryo (A). Scale bars=50 μm. Early embryos (A–C) are indicated by arrowheads; h, heart-shaped embryo; t, torpedo-stage embryo; e, endosperm; s, suspensor. (F) RT–PCR to show *CLE19* expression in ovules and embryos, but not in mixed endosperm and testa tissues (En+Ts). *EIF4A* was used as an internal standard. Control, without cDNA.

To investigate the expression of *CLE19* in *Arabidopsis*, a *pCLE19:GUS:tCLE19* reporter construct was made, with the coding region of *pCLE19:CLE19*_*G6T*_ *:tCLE19* replaced by the *GUS* gene, and transformed into *Arabidopsis* (Col-0). In ovules from the transgenic plants obtained, *GUS* expression was first observed in embryos at the late globular stage (Fig. 2A, B), and persisted in the triangular (Fig. 2C), heart-shaped (Fig. 2D), and torpedo-stage (Fig. 2E) embryos. No *GUS* expression was observed in testa or suspensors.

A weak GUS signal was detected in the ESR (Fig. 2C–E) in *pCLE19:GUS:tCLE19* transgenic plants. To examine if the signal is from expression or diffusion, RT–PCR was performed using RNA extracted from (i) whole ovules; (ii) isolated embryos; and (iii) the mixed endosperm and testa tissues when embryo development in these ovules was at the heart-shaped stage. Expression of the *CLE19* transcript was detected in whole ovules and in embryos, but not in mixed endosperm and testa tissues (Fig. 2F), suggesting that the weak GUS staining signal detected in the ESR was likely to be diffused from the GUS-positive embryo.

**Fig. 3.**
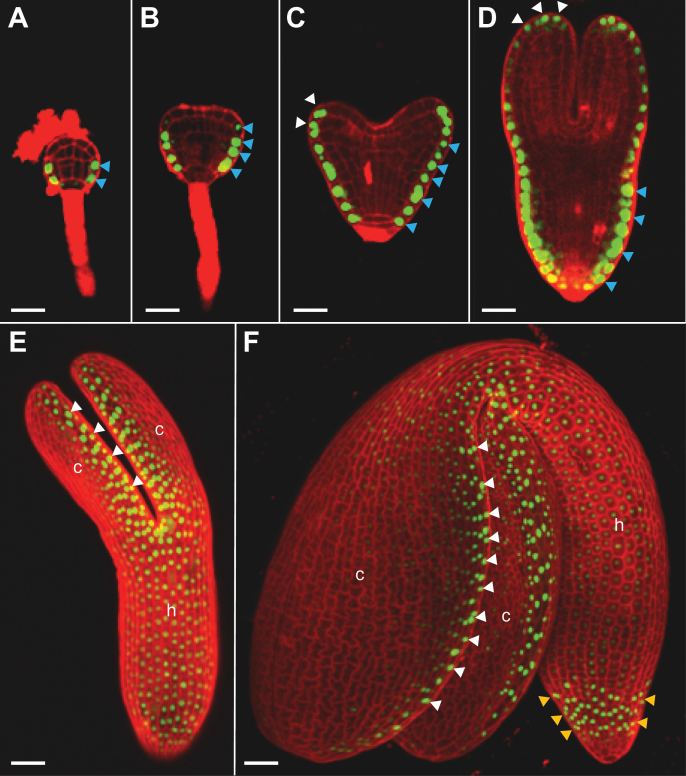
Dynamic expression of *CLE19* during embryogenesis. As examined under a confocal microscope in embryos excised from *pCLE19:SV40-3XGFP:tCLE19* transgenic plants, *GFP* expression was first observed in protodermal cells (indicated by blue arrowheads) at the lower portion of the 32-cell stage embryo (A), and persisted in their progeny cells in the triangular-stage embryo (B). Additional *GFP* expression was observed in epidermal cells at the tips and abaxial sides of the cotyledon (indicated by white arrowheads) in heart-shaped (C) and torpedo-stage embryos (D). In walking-stick (E) and cotyledonary-stage embryos (F), strong *GFP* expression was seen in epidermal cells located at the edges of cotyledons (c, indicated by white arrowheads) and in root caps (indicated by yellow arrowheads), weak *GFP* expression was observed in the hypocotyl (h). (E) and (F) were prepared by superimposing multiple scanned images. Scale bars=50 μm.

A *pCLE19:SV40-3XGFP:tCLE19* reporter construct with a nuclear-localized triple *GFP* (*SV40-3XGFP*) was made and transformed into *Arabidopsis* (Col-0) to define further the expression pattern of *CLE19* in developing embryos. CLSM was performed in embryos dissected from *pCLE19:SV40-3XGFP:tCLE19* transgenic plants. *GFP* expression was first observed in epidermal cells located in the lower portion of the embryo at the late globular stage, and in their progeny cells in triangular-stage embryos (indicated by blue arrowheads; Fig. 3A, B). In heart-shaped and torpedo-stage embryos, additional *GFP* expression was observed in cotyledon primordia (indicated by white arrowheads; Fig. 3C, D). In walking-stick and cotyledonary-stage embryos, *GFP* expression was observed in cells located at the edges of the two cotyledons and cells in the root cap (Fig. 3E, F). This expression pattern differs slightly from that observed previously for *BnCLE19* from *Brassica napus*, as no expression was observed in the hypocotyl and root cap regions when the *BnCLE19* regulatory elements were examined (Fiers *et al.*, 2004).

### Cotyledon establishment, not initiation, is defective in *pCLE19:CLE19*_*G6T*_ *:tCLE19* transgenic plants

**Fig. 4.**
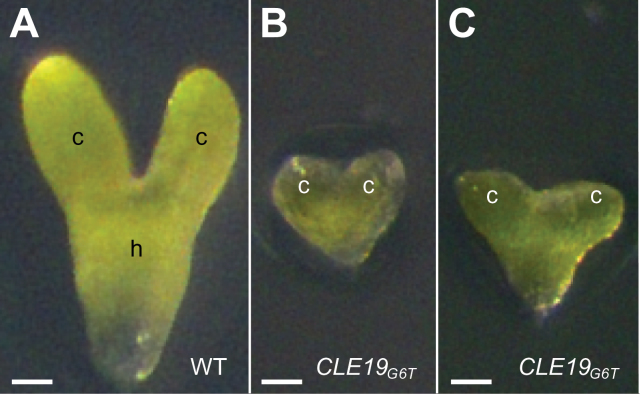
Embryo culture *in vitro* did not rescue the defective cotyledon phenotype in embryos excised from *pCLE19:CLE19*_*G6T*_ *:tCLE19* transgenic plants. Note the abnormal cotyledon establishment in embryos from *pCLE19:CLE19*_*G6T*_ *:tCLE19* transgenic plants (B, C), as compared with the control embryo from the wild type (A). c, cotyledon; h, hypocotyl. Scale bars=50 μm.

The major defect of *pCLE19:CLE19*_*G6T*_ *:tCLE19* transgenic plants was observed in embryos after the heart-shaped stage. Therefore, *in vitro* embryo culture was used to examine whether the defective cotyledon development phenotype could be rescued. Early heart-shaped embryos were isolated from the wild-type and transgenic plants and cultured *in vitro*. After 5 d of cultivation, wild-type embryos had progressed to the cotyledonary stage and had formed two well-established cotyledons (Fig. 4A). However, embryos isolated from *pCLE19:CLE19*_*G6T*_ *:tCLE19* transgenic plants exhibited a severe defect in cotyledon establishment (Fig. 4B–D), suggesting that the cotyledon arrest was intrinsic to the embryo.

**Fig. 5.**
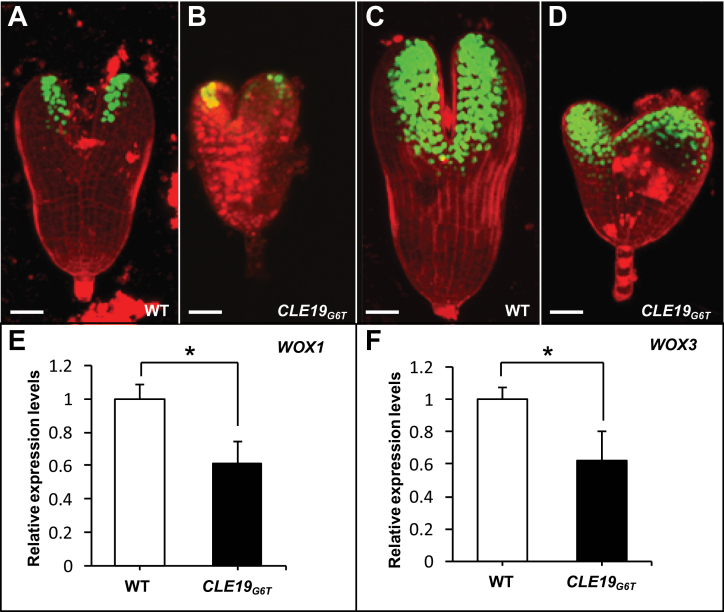
Expression of cotyledon-specific genes in arrested embryos from *pCLE19:CLE19*_*G6T*_ *:tCLE19* transgenic plants. (A–D) Confocal microscopic examinations of embryos excised from wild-type plants (WT) carrying *pWOX1:SV40-3XGFP* (A) or *pWOX3:SV40-3XGFP* marker constructs (C), or from plants carrying *pWOX1:SV40-3XGFP/pCLE19:CLE19*_*G6T*_ *:tCLE19* (B) or *pWOX3:SV40-3XGFP/pCLE19:CLE19*_*G6T*_ *:tCLE19* double constructs (D). Note that similar *GFP* expression, though with a reduced level, was observed in cotyledon primordia of arrested embryos from *pCLE19:CLE19*_*G6T*_ *:tCLE19* transgenic plants (*CLE19*_*G6T*_), as compared with embryos from plants carrying only marker constructs (A, C). Scale bars=50 μm. (E, F) qRT-PCR showed reduced levels of *WOX1* (E) and *WOX3* expression (F) in ovules from the wild type (WT) and *pCLE19:CLE19*_*G6T*_ *:tCLE19* transgenic plants (*CLE19*_*G6T*_). Data represent the mean ±SD from three independently extracted RNA samples. Asterisks indicate significant differences from the wild type (*P* < 0.01 by Student’s *t*-test).

To elucidate further whether the defect in cotyledon development in *pCLE19:CLE19*_*G6T*_ *:tCLE19* plants is attributed to failures in cotyledon initiation or cotyledon establishment, two cotyledon marker lines were developed based on published *in situ* hybridization results for *WUSCHEL-RELATED HOMEOBOX 1* (*WOX1*) and *WOX3* (Haecker *et al.*, 2004). Upstream sequences from *WOX1* and *WOX3* (4454bp and 5028bp, respectively) were fused to the *SV40-3XGFP* reporter gene and transformed into the wild-type *Arabidopsis* (Col-0). Transgenic plants were examined and crossed to *pCLE19:CLE19*_*G6T*_ *:tCLE19* plants, and progeny plants carrying *pCLE19:CLE19*_*G6T*_ *:tCLE19* and homozygous *pWOX1:SV40-3XGFP* or *pWOX3:SV40-3XGFP* constructs were examined under CLSM. As shown in Fig. 5, *GFP* expression in torpedo-stage embryos from wild-type plants carrying *pWOX1:SV40-3XGFP* was restricted to cells located at the edges of two cotyledons (Fig. 5A). Embryos from plants carrying both the *pCLE19:CLE19*_*G6T*_ *:tCLE19* and the *pWOX1:SV40-3XGFP* constructs exhibited a similar *GFP* expression pattern despite a severe delay in cotyledon development in these embryos (Fig. 5B). *GFP* expression in wild-type torpedo-stage embryos carrying the *pWOX3:SV40-3XGFP* construct was observed at the adaxial side of two cotyledons (Fig. 5C). Similar *GFP* expression was also observed in transgenic plants carrying both the *pWOX3:SV40-3XGFP* and *pCLE19:CLE19*_*G6T*_ *:tCLE19* constructs (Fig. 5D). Real-time PCR analyses confirmed that both *WOX1* and *WOX3* were expressed in *pCLE19:CLE19*_*G6T*_ *:tCLE19* plants, albeit with a 30–40% reduction observed in ovules carrying defective embryos compared with those from the wild type (Fig. 5E, F). These data suggest that cotyledon initiation was unaffected in *pCLE19:CLE19*_*G6T*_ *:tCLE19* transgenic plants.

### Endosperm development is delayed in *pCLE19:CLE19*_*G6T*_ *:tCLE19* transgenic plants

**Fig. 6.**
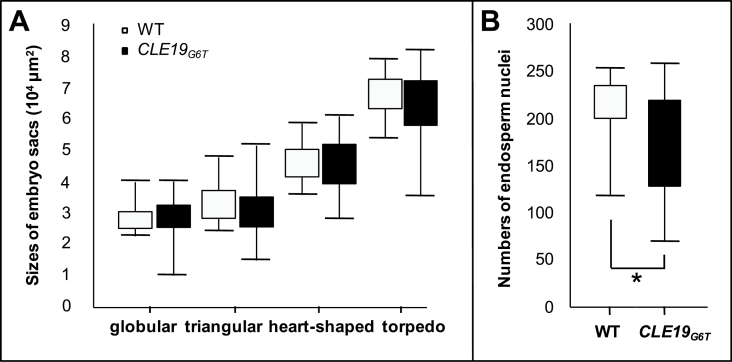
Delayed embryo sac and endosperm development in *pCLE19:CLE19*_*G6T*_ *:tCLE19* transgenic plants. (A) Smaller sizes of embryo sacs in *pCLE19:CLE19*_*G6T*_ *:tCLE19* transgenic plants (*CLE19*_*G6T*_) as compared with those in the wild type (WT). Box plots are used to show total embryo sac areas. The whole area occupied by the embryo and endosperm was measured. Upper and lower bars represent the largest and the smallest sizes of embryo sacs, respectively. Note that smaller embryo sac sizes were observed in transgenic plants at all four stages (globular, triangular, heart-shape, and torpedo) examined (*n*=30). (B) Lower numbers of endosperm nuclei in *pCLE19:CLE19*_*G6T*_ *:tCLE19* transgenic plants (*CLE19*_*G6T*_) compared with the wild type (WT) when counted at the triangular stage of embryo development (*n*=30). Significantly reduced endosperm nuclei numbers (*P*<0.05 by Welch’s *t*-test) were observed in transgenic plants (indicated by asterisks).

To examine further the endosperm defect in *pCLE19:CLE19*_*G6T*_ *:tCLE19* transgenic plants, the size of the embryo sac (the total area occupied by the endosperm and the embryo) was measured in ovules when embryos were at the late globular, triangular, heart-shaped, or torpedo stages. Compared with those in the wild type, embryo sacs in ovules from siliques of *pCLE19:CLE19*_*G6T*_ *:tCLE19* transgenic plants exhibited much greater variation in size. Many embryo sacs from the transgenic plant were smaller than those from the wild type at the same stage (Fig. 6A). The smallest embryo sac in *pCLE19:CLE19*_*G6T*_ *:tCLE19* transgenic plants was only half the size of those from the wild type, while the largest embryo sac was not significantly bigger than those from the wild type (Fig. 6A). This result suggests that endosperm development is delayed in *pCLE19:CLE19*_*G6T*_ *:tCLE19* transgenic plants.

Next, the number of endosperm nuclei was counted in ovules from the wild-type and *pCLE19:CLE19*_*G6T*_ *:tCLE19* transgenic plants. A highly variable number of nuclei was observed in ovules from *pCLE19:CLE19*_*G6T*_ *:tCLE19* transgenic plants when embryos inside these ovules were at the triangular stage. In general, the number of endosperm nuclei in these transgenic plants was significantly lower than that in the wild type (Fig. 6B), which was consistent with the smaller embryo sac observed in transgenic plants.

### Expression of early endosperm-specific genes is prolonged in ovules from *pCLE19:CLE19*_*G6T*_ *:tCLE19* transgenic plants

**Fig. 7.**
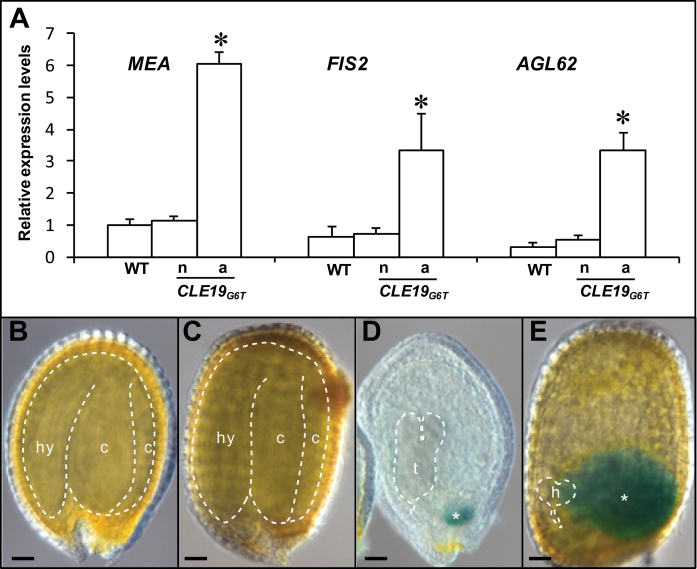
Prolonged and elevated expression of early endosperm-specific genes in the endosperm of *pCLE19:CLE19*_*G6T*_ *:tCLE19* transgenic plants. (A) qRT-PCR analyses showed elevated expression of *MEA*, *FIS2*, and *AGL62* in those abnormal ovules (a) from a *pCLE19:CLE19*_*G6T*_ *:tCLE19* transgenic plant (*CLE19*_*G6T*_), as compared with those normal ovules (n) from the same plant, and ovules from the wild type (WT). All ovules were analysed at 12 DAP. Data represent the mean ±SD of three independently extracted RNA samples. (B–E) *GUS* expression in seeds from the wild type (B) and *pCLE19:CLE19*_*G6T*_ *:tCLE19* transgenic plants carrying a *pMEA:GUS* reporter construct, examined at 12 DAP (C–E). Note that aborted seeds in (D) and (E) showed prolonged *GUS* expression in chalarzal endosperm (marked by asterisks), but not in the normal seed (C) in the same plant. Embryos are traced with dotted lines. c, cotyledon; hy, hypocotyl; h, heart-shaped stage embryo; t, torpedo-stage embryo. Asterisks indicate significant differences from the wild-type (*P*<0.01 by Student’s *t*-test). Scale bars in B–E=50 μm.

To determine whether expression of the early endosperm-specific genes was altered in *pCLE19:CLE19*_*G6T*_ *:tCLE19* transgenic plants, expression of *MEA*, *FIS2*, and *AGL62*, which are expressed in the endosperm prior to cellularization (Luo *et al.*, 2000; Kang *et al.*, 2008), was analysed at 10–12 DAP using qRT-PCR. Compared with the wild-type, expression of all these three genes was elevated in those abnormal ovules from *pCLE19:CLE19*_*G6T*_ *:tCLE19* transgenic plants (Fig. 7A). However, no significant difference in expression levels was seen between wild-type ovules and those normal-looking ovules from the transgenic plants (Fig. 7A).

To access the *MEA* expression pattern in the *pCLE19:CLE19*_*G6T*_ *:tCLE19* transgenic plants, a *pMEA:GUS* reporter construct (Luo *et al.*, 2000) was introduced into *pCLE19:CLE19*_*G6T*_ *:tCLE19* transgenic plants by crossing. Similar *GUS* expression was observed in endosperms from the wild type and *pCLE19:CLE19*_*G6T*_ *:tCLE19* transgenic plants in the first 5 DAP, and no *GUS* expression was detected in those wild-type ovules or wild-type-appearing ovules from *pCLE19:CLE19*_*G6T*_ *:tCLE19* transgenic plants at 12 DAP (Fig. 7B, C). However, in those aborted seeds from the transgenic plants, *GUS* expression was often observed in the chalarzal region of the endosperm (Fig. 7D, E), suggesting a prolonged *MEA* expression in chalarzal endosperms in aborted seeds. This observation provides further evidence for delayed endosperm development in *pCLE19:CLE19*_*G6T*_ *:tCLE19* plants.

### Expression of *CLE19*_*G6T*_ in the ESR of the endosperm mimics the defective cotyledon phenotype in *pCLE19:CLE19*_*G6T*_ *:tCLE19* transgenic plants

The promoter from *ALE1*, expressed in the ESR of the endosperm in *Arabidopsis* (Tanaka *et al.*, 2001), was used to examine if ectopic expression of *CLE19*_*G6T*_ in the endosperm can cause a defect in the embryo. To test the specificity of the *ALE1* promoter, the *ALE1* upstream region (1875bp) was fused to the *SV40-3XGFP* reporter gene to create *pALE1:SV40-3XGFP* and transformed into *Arabidopsis* (Col-0). As expected, *GFP* expression was observed specifically in the ESR of endosperms, and no *GFP* expression was observed in embryos (Supplementary Fig. S1 at *JXB* online).

**Fig. 8.**
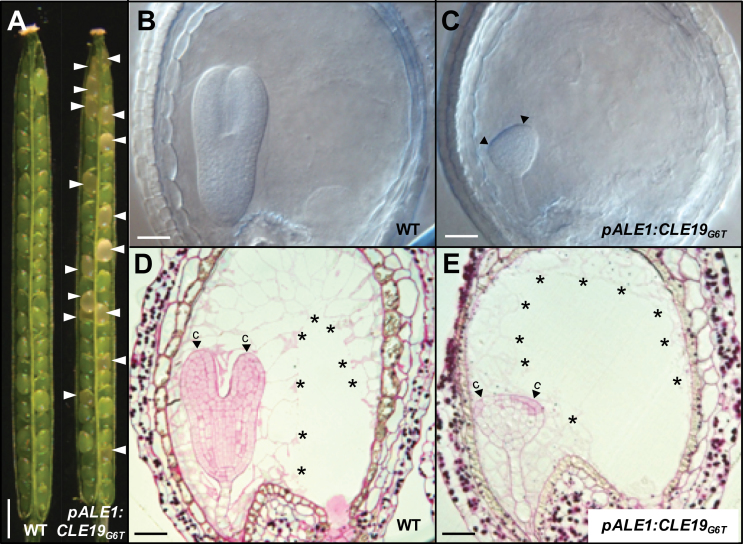
Endosperm-specific expression of *CLE19*_*G6T*_ led to defective cotyledon establishment in embryos. (A) Seed abortions (indicated by arrowheads) observed in the *pALE1:CLE19*_*G6T*_ transgenic plant, as compared with the wild type (WT). (B and C) Defected cotyledon establishment (indicated by arrowheads) in embryos from *pALE1:CLE19*_*G6T*_ transgenic plants (C), as compared with the wild type (B) at the same stage (7 DAP). (D and E) Cytohistological examination of ovules from the wild type (D) and *pALE1:CLE19*_*G6T*_ transgenic plants (E), showing establishment of the defective cotyledon (E, indicated by arrowheads). Note the cellularized endosperms (indicated by asterisks). c, cotyledon primordia. Scale bars: in A=1mm; in B–E=100 μm.

Next, a *pALE1:CLE19*_*G6T*_ construct was made and transformed into *Arabidopsis* (Col-0). Among the 35 individual transgenic lines obtained, three (#1, #2, and #3) showed high ratios of seed abortion (indicated by arrowheads; Fig. 8A), with abortion frequencies of 33.3, 28.4, and 32.9%, respectively (Table 1). The phenotypes of these lines were similar to one another, and line #1 was selected for further analyses. Examination under a DIC microscope showed that embryo development in the aborted seeds was mostly arrested at the triangular stage (Fig. 8B, C), slightly earlier than observed in *pCLE19:CLE19*_*G6T*_ *:tCLE19* transgenic plants. These enlarged abnormal triangular embryos were often asymmetrical, most probably as a result of defective cotyledon development (indicated by arrowheads; Fig. 8C). Semi-thin sections in combination with PAS staining showed that endosperm cellularization occurred relatively normally in these aborted seeds (indicated by asterisks; Fig. 8D, E), while cotyledon development was severely arrested (indicated by arrowheads; Fig. 8C, E). The cell division pattern in the lower portion of the arrested embryos was normal, with a well-formed hypophysis and suspensor (Fig. 8E). It seems that expression of *CLE19*_*G6T*_ under the control of the *ALE1* promoter led to an earlier defect in cotyledon establishment in embryos than that observed in *pCLE19:CLE19*_*G6T*_ *:tCLE19* transgenic plants, and no evident defect in endosperm development. These data indicate that the *CLE19*_*G6T*_ expressed in the endosperm interferes with embryo development in a non-cell-autonomous manner.

## Discussion

Genetic studies in *Arabidopsis* have identified a large number of *embryo-defective* (*emb*) mutants that exhibit a spectrum of embryo-lethal phenotypes, and endosperm development in these mutants is usually also defective (Liu and Meinke, 1998; McElver *et al.*, 2001; Muralla *et al.*, 2011), demonstrating indirectly that embryo and endosperm development are tightly linked. *ALE1*, *ZOU*, *IKU2*, and *ESF1* are only expressed in endosperms, not in embryos; mutations or down-regulation of these genes cause defects in embryos (Tanaka *et al.*, 2001; Garcia *et al.*, 2003; Xing *et al.*, 2013; Yang *et al.*, 2008; Costa *et al.*, 2014), suggesting that endosperm-expressed genes are involved in plant embryogenesis. In contrast, there was no direct evidence to show that embryo-expressed genes regulate endosperm development. In this study, it was shown that *CLE19*, as an embryo-expressed *CLE* gene, regulates both embryo and endosperm development in *Arabidopsis*.

In recent years it has been shown that *CLE* genes function as small extracellular peptides to regulate a number of developmental processes in a non-cell-autonomous manner (Fletcher *et al.*, 1999; Fiers *et al.*, 2005; Murphy *et al.*, 2012). Two *CLE* genes are implicated in embryo development. *CLE8*, which is expressed in the embryo proper and the endosperm, regulates suspensor divisions via activation of *WOX8* expression (Fiume and Fletcher, 2012). *BnCLE19* was first identified in *B. napus* as a *CLE* gene expressed in cotyledon primordia during embryogenesis (Fiers *et al.*, 2004). Overexpression of *BnCLE19* and treatment of wild-type *Arabidopsis* seedlings with 14 amino acid CLE19 peptides led to *CLV2*-dependent premature stem cell differentiation in the root meristem (Fiers *et al.*, 2004, 2005). Since the T-DNA insertional *cle19* mutant in *Arabidopsis* showed no visible phenotype (Fiers *et al.*, 2004), the function of the endogenous *CLE19* remains unknown. Expression analyses performed in this study revealed that, in addition to those epidermal cells in cotyledon primordia as in *BnCLE19* (Fiers *et al.*, 2004), *CLE19* in *Arabidopsis* was also expressed in epidermal cells of hypocotyls and root caps, while no expression was observed in the endosperm. Further studies showed that transgenic plants carrying the *pCLE19:CLE19*_*G6T*_ *:tCLE19* construct exhibited a dominant seed abortion phenotype, with defective cotyledon establishment in embryos and compromised nuclear proliferation and cellularization in endosperms, suggesting a role for CLE19 in co-ordinating embryo and endosperm development. This hypothesis was further supported by the observation that the defective cotyledon establishment phenotype in *pCLE19:CLE19*_*G6T*_ *:tCLE19* transgenic plants was mimicked when *CLE19*_*G6T*_ was expressed under the control of an endosperm-specific *ALE1* promoter. It has been reported before that synthetic 14 amino acid CLE19 peptide applied to seedlings *in vitro* promotes stem cell differentiation in root meristems (Fiers *et al.*, 2005; Tamaki *et al.*, 2013). Most probably CLE19 peptides produced in embryos act as a differentiation-promoting signal to regulate cotyledon establishment in embryos, and to promote nuclear division and cellularization in endosperms. Further studies are needed to identify receptors involved in perceiving the CLE19 peptide.

The formation of cotyledons is a milestone event in embryo development, marking the initiation of a major organogenesis phase in the embryo. Auxin biosynthesis and transport are involved in cotyledon initiation, determining the emergence and the positions of cotyledon primordia (Liu *et al.*, 1993; Furutani *et al.*, 2004; Cheng *et al.*, 2007). In addition, several *WOX* family transcription factors are expressed during cotyledon formation: *WOX1* and *WOX3* are expressed at positions where the two cotyledon primordia are formed (Haecker *et al.*, 2004), while *WOX2* is expressed in the apical region of the globular stage embryos (Haecker *et al.*, 2004). Although the *wox1/wox3* double mutant exhibited no defect in cotyledon formation, *wox1/wox2*/*wox3* triple mutations enhanced the *wox2* defect in cotyledon formation (Breuninger *et al.*, 2008), suggesting a synergic interaction among these transcription factors. *CUP-SHAPED COTYLEDON 1* (*CUC1*) and *CUC2* are expressed in the boundary of two cotyledons and regulate the final patterning of cotyledons (Aida *et al.*, 1997, 1999; Vroemen *et al.*, 2003), while *ASYMMETRIC LEAVES 1* (*AS1*) and *AS2* regulate the adaxial–abaxial pattern of the cotyledons (Byrne *et al.*, 2000; Lin *et al.*, 2003). Data from this study showed that the primary defect of *pCLE19:CLE19*_*G6T*_ *:tCLE19* transgenic plants is the establishment, not the initiation, of these cotyledons, suggesting a multilayered regulation of cotyledon formation.

It is also interesting to note that only a fraction of seeds produced in *pCLE19:CLE19*_*G6T*_ *:tCLE19* and *pALE1:CLE19*_*G6T*_ transgenic plants showed defects in embryo development; the remaining embryos are able to form normal seeds. Such an incomplete penetration of phenotypes has been reported in overexpression or mutations of several *CLE* genes. For example, when *BnCLE19* was overexpressed under the control of the CaMV *35S* promoter, only 17% of the transgenic plants obtained exhibited the short-root and pin-shaped pistil phenotypes (Fiers *et al.*, 2004). In the homozygous *cle8-1* mutant (with a point mutation in the CLE motif), only 15% of embryos showed defects in embryogenesis (Fiume and Fletcher, 2012). It is believed that dosage effects, regulated most probably at the post-transcriptional peptide processing levels, are very important for peptide hormones.

There is no doubt that the antagonistic peptide technology provides a powerful *in vivo* tool to study the function of *CLE* genes (Song *et al.*, 2013). The technology has already been used to elucidate the role of *CLE22* in root development (Song *et al.*, 2013), and *CLE45* in phloem differentiation (Rodriguez-Villalon *et al.*, 2014). The phenotypic and genetic analyses of the *CLE19* gene in *Arabidopsis* by expression of the *CLE19*_*G6T*_ construct under control of the endogenous *CLE19* promoter or the endosperm-specific *ALE1* promoter in this work allowed the roles of *CLE19* in co-ordinating embryo and endosperm development to be defined. When endogenous regulatory elements are used in this type of study, it is expected that the antagonistic peptides produced may act *in situ* to interfere with the signal transduction pathways involved. One study published in this issue showed that synthetic CLE peptides with a G6T substitution are less effective than wild-type peptides in seedling treatments *in vitro* (Czyzewicz *et al.*, 2015). This is expected since it has been shown in CLV3 that the G6 residue is critical for the CLV3 function: (i) a *clv3-1* mutant with a severe defect in SAM homeostasis was caused by a substitution in G6 (Fletcher *et al.*, 1999); (ii) *pCLV3:CLV3*_*G6A*_ *:tCLV3* is one of the least effective constructs in complementing the *clv3-2* mutant (Song *et al.*, 2012); and (iii) no transgenic plants carrying the *pCLV3:CLV3*_*G6T*_ *:tCLV3* construct showed complete complementation of the *clv3-2* phenotype (Song *et al.*, 2013). Although it has been shown that treatment of wild-type *Arabidopsis* seedlings with CLV3_G6T_ peptide *in vitro*, with peptides being refreshed every day, caused a slightly enlarged SAM phenotype (a weak *clv3*-like phenotype; Song *et al.*, 2013), whether the antagonistic peptide technology could be used effectively *in vitro* remains to be evaluated further.

In summary, the results obtained in this study suggest that the *CLE19* expressed in cotyledon primordia of *Arabidopsis* may act as a peptide ligand that constitutes a diffusible signal to regulate cotyledon establishment in embryos and to promote the nuclear proliferation and cellularization in endosperms.

## Supplementary data

Supplementary data are available at *JXB* online

Figure S1. *ALE1* promoter activity in seed development.

Table S1. Primers used in this study.

Supplementary Data
