# Supplementary material for: CLE19 expressed in the embryo regulates both cotyledon establishment and endosperm development in Arabidopsis
Source: J Exp Bot. 2015 Jun 12;66(17):5217–27. doi: 10.1093/jxb/erv293 (PMC4526921; doi:10.1093/jxb/erv293)
Supplement: Supplementary Data [file supp_66_17_5217__index.html]

 CLE19 expressed in the embryo regulates both cotyledon establishment and endosperm development in Arabidopsis — CLE19 expressed in the embryo regulates both cotyledon establishment and endosperm development in Arabidopsis — Supplementary Data 

# *CLE19* expressed in the embryo regulates both cotyledon establishment and endosperm development in *Arabidopsis*

## Supplementary Data

Data files

- Supplementary Data - Supplementary Data
- Supplementary Data - Supplementary Data
