# Supplementary material for: CLE19 expressed in the embryo regulates both cotyledon establishment and endosperm development in Arabidopsis
Source: J Exp Bot. 2015 Jun 12;66(17):5217–27. doi: 10.1093/jxb/erv293 (PMC4526921; doi:10.1093/jxb/erv293)
Supplement: Supplementary Data [file supp_erv293_jexbot149294_file002.pdf]

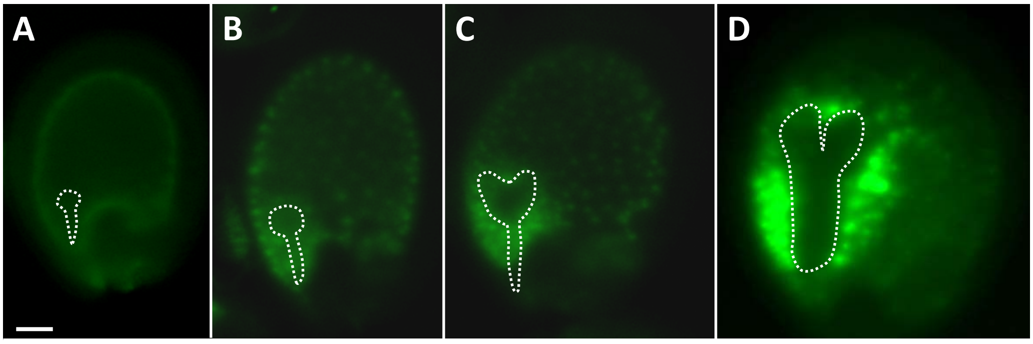

Supplementary Fig. 1. *ALE1* promoter activity in seed development.
GFP expression was observed in ESR of ovules when embryos inside were at the late globular (B), heart-shaped (C) and torpedo stages (D) in transgenic plants carrying the *pALE1:SV40-3×GFP* construct. No *GFP* expression was observed in ovule when the embryo inside was at the globular stage (A).
