# Supplementary material for: CLE19 expressed in the embryo regulates both cotyledon establishment and endosperm development in Arabidopsis
Source: J Exp Bot. 2015 Jun 12;66(17):5217–27. doi: 10.1093/jxb/erv293 (PMC4526921; doi:10.1093/jxb/erv293)
Supplement: Supplementary Data [file supp_erv293_jexbot149294_file001.pdf]

Supplementary Table S1. Primers used in the study

Name	Sequence
**CLE19attB1**	GGGGACAAGTTTGTACAAAAAAGCAGGCTCTAACCTTTTAGTT
**CLE19attB2**	GGGGACCACTTTGTACAAGAAAGCTGGGTAGAATACTGATTGT
**CLE19mtF**	TCCCACTACTCCAAATCCACTCCACAAC
**CLE19mtR**	GTGGATTTGGAGTAGTGGGAATTACCCG
**AdapF-CLE19pro-FP**	TAGTTGGAATGGGTTCGAACTAACCTTTTAGTTGTAAAGCTAG
**AdapR-CLE19pro-RP**	TTATGGAGTTGGGTTCGAATTGTCTATTTTTGGTCAAATATTGT
**AdapF-WOX1pro-FP**	TAGTTGGAATGGGTTCGAATGCCAGCAAAAATTTCACAG
**AdapR-WOX1pro-RP**	TTATGGAGTTGGGTTCGAATTTGGTGTGTACTTAATTTATATGTATG
**AdapF-WOX3pro-FP**	TAGTTGGAATGGGTTCGAATGTAGAACGATTGACGTATCGG
**AdapR-WOX3pro-RP**	TTATGGAGTTGGGTTCGAATCTCCGTTCAGACAAATATTGATG
**AdapF-ALE1pro-FP**	TAGTTGGAATGGGTTCGAATCCTCACTACTTCATAGAGCTG
**AdapR-ALE1pro-RP**	TTATGGAGTTGGGTTCGAATAGATCTTTATTAGGATTCTCCATTTG
**tCLE19-NosTer-RP**	CATATAATTGTTCTTCAATTACCACAGTTTGAACGATCGGGGATC
**NosTer-tCLE19-FP**	GATCCCCGATCGTTCAAACTGTGGTAATTGAAGAACAATTATATG
**AdapR-tCLE19-RP**	TTATGGAGTTGGGTTCGAAAGAATACTGATTGTTATCCGGC
**CLE19cds-ALE1pro-RP**	CATCAAACCCTTTATCTTCATTAGATCTTTATTAGGATTCTCCATTTG
**ALE1pro-CLE19cds-FP**	CAAATGGAGAATCCTAATAAAGATCTAATGAAGATAAAGGGTTTGATG
**AdapR-CLE19-RPstop**	TTATGGAGTTGGGTTCGAATTACCTGTTGTGGAGTGG
**EIF4A-RT-FP**	GGAACACAATTTGATACGCG
**EIF4A-RT-RP**	GTCAAGCTGCTGCAAGAC
**CLE19-FP**	ATGAAGATAAAGGGTTTGATG
**CLE19-RP**	TTACCTGTTGTGGAGTGGA
**EIF4A-FP**	TGACCAGAGGCTGAATGAAGT
**EIF4A-RP**	CGTAAGCATAGATACCCCTAAGAA
**WOX1-FP**	CCACGGATTCGACAAGAAAGATC
**WOX1-RP**	TCGTCTCTCGTCTCCTCCAC
**WOX3-FP**	ATCCTTCTCCCATGTGTCTTCC
**WOX3-RP**	ATGAGGGATATGATGGTCGTGG
**MEA-FP**	TGGGCAGGACTATGGTTTGG
**MEA-RP**	CCGAAACATCCACTTCGAGGTA
**FIS2-FP**	GGGTTCTAAAGGATGATGTAGG
**FIS2-RP**	CGAGGTAAGTCATCTAAATCCA
**AGL62-FP**	CGAAATTCGATTGTTCAGGATC
**AGL62-RP**	CAAGGGCTTTTGTCTTTTCTC
